# Methods in food defence: a simple and robust screening for 16 rodenticides by LC-DAD/FLD following QuEChERS–based extraction

**DOI:** 10.1007/s00216-022-04145-w

**Published:** 2022-06-17

**Authors:** Georg Menacher, Benedikt Masberg, Paul W. Elsinghorst

**Affiliations:** 1 Central Institute of the Bundeswehr Medical Service Munich, Ingolstädter Landstraße 102, 85748 Garching, Germany; 2 Pharmaceutical Institute, University of Bonn, 53121 Bonn, Germany; 3 Institute of Nutrition and Food Sciences, University of Bonn, 53115 Bonn, Germany

**Keywords:** Food defence, Rodenticides, QuEChERS, LC-DAD, LC-FLD

## Abstract

Fortunately, the intentional contamination of food or water supplies out of criminal or terroristic motivation is a rather rare event. However, in the face of asymmetric warfare and as the consequences of such an event would be severe, food defence as a necessary supplement to food safety is gaining increased attention. While some progress has been made in developing non-target detection devices, the contamination of food or water supplies using readily available rodenticides may still be revealed only by complex analytical techniques. The presented study therefore aimed to develop a quick and easy screening method for the detection of sixteen globally common rodenticides in foodstuffs. Robust operation with limited personnel and analytical resources were one benchmark to be met by the method, which uses a slightly modified QuEChERS (quick, easy, cheap, effective, rugged, safe) protocol for dispersive solid-phase extraction and subsequent ion-pair chromatography with diode-array and fluorescence detection. Quantification limits were as low as 5 µg/kg with satisfying bias (recovery) and repeatability rates of 77 to 117% and 1.8 to 17.1%, respectively. The developed method provides reliable and robust detection of these deadly poisons at toxic concentrations, which was demonstrated impressively in an improvised assault scenario.

**Supplementary Information:**

The online version contains supplementary material available at 10.1007/s00216-022-04145-w.

## Introduction

Attacks on public health by poisoning of water wells or grain stocks were common means in ancient warfare. But as modern weapons developed, such insidious assaults became virtually obsolete and for centuries poisoning of food or water supplies was attributed only to criminal activities like murder or blackmail. However, terrorist attacks and asymmetric warfare have raised public awareness that food and water supplies must be secured against intentional contamination by food terrorism [1]. Activities in this respect are generally summarized under the term food defence, which must be carefully distinguished from other common terms like food fraud or food safety, where food safety covers all aspects of unintentional contamination and food fraud any adulteration for economic gain but without harm [2, 3].

Today, public food control authorities still concentrate on food safety and counteracting food fraud, whereas food defence is mostly left to food industries. Current food production standards recognized by the Global Food Safety Initiative like IFS Food or BRCGS follow the established farm to fork strategy and focus on preventive measures along the supply chain [4]. Although the proposed actions and requirements appear reasonable in the context of food production, insufficient guidance remains for analytical methods that can detect a malicious contamination despite all defensive means. Such intentional contamination may use biological or chemical agents and sometimes may require only very low amounts [5, 6]. While detection techniques for most of the possibly deployed bacteria or viruses do already exist, methods for many of the chemical toxins are still lacking, especially in the context of food or water contamination.

Projects like EDEN or SNIFFER as part of the EU Seventh Framework Program tried to design and evaluate new detection tools for a variety of food contamination scenarios but only with marginal success [7]. Even the limited and somewhat arbitrary selection of toxicants (mercury chloride, methylmercury, bromadiolone, sodium trifluoracetate, triacetone triperoxide, quaternary ammonium compounds, pyrrolizidine alkaloids, cereulide, *Bacillus anthracis*, *Bacillus cereus*) revealed that the design of one universal detection device appears almost impossible.

However, one of the compounds used in project EDEN was bromadiolone, a potent rodenticide belonging to the group of superwarfarins that are readily available around the globe in kilogram quantities and of insidious delayed toxicity. Small amounts of these deadly substances last to poison large populations, especially because of their late onset of symptoms leading if untreated to haemorrhagic bleeding and potentially death [8]. Put at hands of terrorists and released into the food supply chain or water supply network, serious harm to public health may result [9, 10]. In the face of this threat, not only must continuous food defence measures be applied, but analytical techniques must also be developed to reveal any successful assault.

Current methods for the detection of rodenticides mainly focus on human and animal samples like blood, serum, urine, or hair in a toxicological context [11]. More recently, also environmental exposure to rodenticides of wildlife has gained attention [12–14]. But analysis of rodenticides in food is still uncommon, and only few reports have been published, mostly addressing a possible carry-over or even poisoning from food or feed [15]. The majority of these methods applies liquid chromatography coupled to mass spectrometry (LC–MS/MS), which may be considered the current state of the art in modern food safety laboratories [16–19]. Older reports using liquid chromatography with UV/Vis (LC-DAD) or fluorescence detection (LC-FLD) have also addressed mainly biological samples, but there are some examples where actually food samples were analysed for one or several rodenticides [20, 21]. Table 1 provides a comparison of three reports addressing chromatographic analysis of food for two common rodenticides, i.e., warfarin, bromadiolone and brodifacoum, by different detection techniques.

**Table 1:** Limits of detection (LOD) and quantification (LOQ) of warfarin (2), bromadiolone (12) and brodifacoum (15) achieved in food by different chromatographic techniques

	LC-DAD [20]		LC-FLD [15]		LC–MS/MS [19]	
Rodenticide	LOD (µg/kg)	LOQ (µg/kg)	LOD (µg/kg)	LOQ (µg/kg)	LOD (µg/kg)	LOQ (µg/kg)
Warfarin (**2**)	2	6.2	31.4	95.2	2.9–4.0	10–13
Bromadiolone (**12**)	5	13	15.7	47.6	2.9–7.5	10–25
Brodifacoum (**15**)	–	–	13.9	42.1	3.2–6.5	11–22

**Fig. 1:**
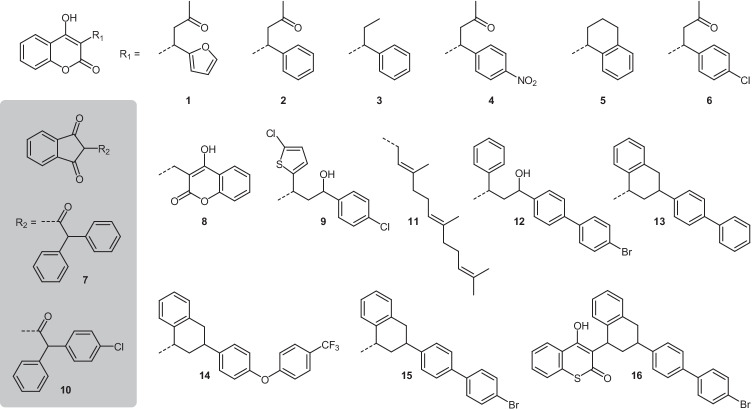
Structures of the investigated rodenticides being either 4-hydroxycoumarins (*n* = 14) or 1,3-indandiones (*n* = 2), which were selected from a literature survey focusing on global availability

As of today, thousands of soldiers are serving in field operations or missions abroad. Although no intentional contamination of food or water supplies by rodenticides has been reported in recent military context, this is a realistic scenario of asymmetric warfare. Currently, NATO has adopted similar food defence instruments as aforementioned industry standards, but also focusing on preventive actions [22]. To additionally provide robust and reliable detection capabilities to deployed laboratories, we here report a quick and easy LC-DAD/FLD–based method for the screening of sixteen globally common rodenticides in water or foodstuffs (Fig. 1).

## Materials and methods

### Chemicals and reagents

LC–grade methanol and acetonitrile were supplied by VWR International (Darmstadt, Germany). Chloroform, acetic acid and sodium hydroxide were acquired from Merck-Millipore (Darmstadt, Germany). Diisopropylethylamine (DIPEA) was purchased from Carl Roth (Karlsruhe, Germany). LC–grade water was prepared in-house using a Milli-Q^®^ Gradient water purification system from Merck (Darmstadt, Germany). Chromabond^®^ QuEChERS extraction mix XII (4 g magnesium sulphate, 1 g sodium chloride) and clean-up mix VI (0.9 g magnesium sulphate, 0.15 g primary secondary amine, 150 mg C_18_) were obtained from Macherey–Nagel (Düren, Germany). Chlorophacinone (CAS: 3691-35-8), coumachlor (CAS: 81-82-3), coumafuryl (CAS: 117-52-2), dicoumarol (CAS: 66-76-2), difethialone (CAS: 104653-34-1), diphacinone (CAS: 82-66-6) and phenprocoumon (CAS: 435-97-2) were purchased from LGC (Augsburg, Germany). Brodifacoum (CAS: 56073-10-0, isomer ratio *cis*/*trans*: 58/41), bromadiolone (CAS: 28772-56-7, isomer ratio *cis*/*trans*: 72/18), coumatetralyl (CAS: 5836-29-3), difenacoum (CAS: 56073-07-5, isomer ratio *cis*/*trans*: 53/46), flocoumafen (CAS: 90035-08-8, isomer ratio *cis*/*trans*: 21/79) and warfarin (CAS: 81-81-2, racemate) were from Sigma-Aldrich (Schnelldorf, Germany). Acenocoumarol (CAS: 152-72-7) and ferulenol (CAS: 6805-34-1) were acquired from Cayman Chemicals (Ann Arbor, MI, USA). Tioclomarol (CAS: 2619-35-8) was obtained from Toronto Research Chemicals (Toronto, Canada).

Stock solutions (1000 µg/mL, 10 mL) were prepared in chloroform (dicoumarol), methanol (difethialone, ferulenol, tioclomarol) or acetonitrile. To achieve sufficient dissolution of difethialone, 0.1 M aqueous sodium hydroxide was added dropwise to the methanolic mixture.

### Sample preparation

Water (10 mL) and acetonitrile (10 mL) were added to the sample (5 g, wheat flour for method development) placed in a 50-mL polypropylene tube and mixed by vigorous shaking for 1 min. After addition of acetic acid (100 µL) and vigorous shaking for 1 min, the QuEChERS extraction mix XII was added and the mixture again vigorously shaken for 1 min. Centrifugation (3000 × *g*, room temperature, 10 min) provided a clear organic supernatant of which 6 mL was transferred to a 15-mL polypropylene tube containing the QuEChERS clean-up-mix VI. The mixture was shaken vigorously (30 s) and centrifuged (3000 × *g*, room temperature, 10 min), and 1 mL of the organic supernatant was transferred to an amber, silanized glass vial. An optional solvent exchange may be carried out by evaporation and subsequent reconstitution using 10 mmol/L acetic acid/DIPEA in a water/methanol mixture (52/48, *v*/*v*) as the acetonitrile from the extraction solution may impair column equilibration and retention time stability.

### LC-DAD/FLD analysis

Sample analysis was performed using an Infinity 1200 series liquid chromatography system (Agilent Technologies, Waldbronn, Germany) consisting of an autosampler, a degasser, a binary pump and a column thermostat coupled to a diode-array (DAD) and fluorescence detector (FLD). Samples (20 µL) were separated at 40 °C using a Kinetex^®^ Biphenyl column (150 mm × 4.6 mm, 2.6 µm; Phenomenex, Aschaffenburg, Germany) protected by a precolumn (SecurityGuard Ultra cartridge for UHPLC Biphenyl; Phenomenex) applying gradient elution (solvent A: 10 mmol/L acetic acid/DIPEA in water, solvent B: 10 mmol/L acetic acid/DIPEA in methanol, flow rate: 0.7 mL/min, min/% B: 0/48, 10/78, 25/98, 30/48). The following parameters were used for detection: DAD, 310 nm, bandwidth 5 nm; FLD, excitation 310 nm, emission 390 nm, PMT gain 10. Data acquisition, processing and analysis were performed using the ChemStation software (Rev. B.04.03, Agilent Technologies).

### Method validation

Linearity was evaluated by linear regression and residual analysis of calibration curves obtained in 10 mmol/L acetic acid/DIPEA in a water/methanol mixture (52/48, *v*/*v*) at 10 different analyte mass concentrations ranging from 0.0001 to 1 µg/mL (coumatetralyl), 0.001 to 10 µg/mL (brodifacoum, bromadiolone, coumachlor, coumafuryl, difenacoum, ferulenol, flocoumafen, phenprocoumon, tioclomarol, warfarin) or 0.01 to 100 µg/mL (acenocoumarol, chlorophacinone, dicoumarol, difethialone, diphacinone). Following EU guidance and in accordance with ISO 11843–2:2000 [23], wheat flour was used as a blank matrix to prepare 10 independent, non-spiked samples following the developed preparation protocol followed by analysis. Limits of detection (LOD) and quantification (LOQ) were subsequently calculated from these tenfold blank experiments and the obtained linear regression parameters by applying Eqs. 1 and 2 with ${s}_{y,b}$: standard deviation of the blank measurements and $b$: slope of the calibration curve:

1 $${\text{LOD}}=\text{3.9}\cdot \frac{{s}_{y,b}}{b}$$

2 $${\text{LOQ}}=\text{3.3}\cdot {\text{LOD}}$$

Bias (recovery) and repeatability were subsequently derived by spiking 8 independent samples of the wheat flour blank matrix to the individual LOQ obtained for each rodenticide and subjecting these samples to sample preparation and analysis.

## Results and discussion

### Optimized QuEChERS conditions

To keep sample preparation as simple as possible while providing sufficient matrix removal for subsequent LC-DAD/FLD analysis, we considered following a QuEChERS–based approach, which is a well-established extraction and clean-up procedure for the analysis of pesticide residues and other contaminants in food and feed [24, 25]. Main advantages of this method are ease of handling, low material costs and high stability of the used reagents, which generally outweigh the possible drawback of obtaining extracts with sometimes high matrix load. Moreover, the QuEChERS method has already been used for the analysis of rodenticides in animal blood and liver tissue [26]. Optimization of the preparation procedure showed that extraction mix XII and clean-up-mix VI provided extracts most suitable for our purposes (data not shown). To achieve sufficient recovery rates, the extraction mixture had to be acidified by acetic acid.

### Chromatographic analysis

In forensic analysis of rodenticides, a standard reversed-phase C_18_ column is commonly applied [11, 26–29]. However, most of the published methods do not cover such a broad selection of rodenticides as in this study or apply mass spectrometry for detection, which does not require baseline separation of all analytes as in the case of LC-DAD/FLD. Therefore, an initial column screening (C_18_, phenylhexyl, pentafluorophenyl, biphenyl, graphite) was carried out and suggested that a biphenyl column provides significantly improved selectivity for sufficient separation. While 4-hydroxycoumarins generally provide well-shaped peaks, 1,3-indandiones are known to show severe peak tailing [17]. This observation may be attributed to keto-enol tautomerism of the 1,3-diketone moiety, which depends on the solvent present and the substitution pattern of the 1,3-indandione ring system [30, 31]. Protic solvents as in reversed-phase liquid chromatography and conjugated acyl substituents as in diphacinone (**7**) or chlorophacinone (**10**) will promote enolization and subsequent dissociation, which leads to peak tailing. This drawback may either be overcome by formation of stable ion pairs or by pH adjustment to a level, where dissociation is shifted completely to one species, e.g., by full protonation under acidic conditions. However, as fluorescence may be impaired or even quenched by protonation, we opted for ion-pair chromatography, which has successfully been applied to rodenticide analysis before [32–34].

**Fig. 2:**
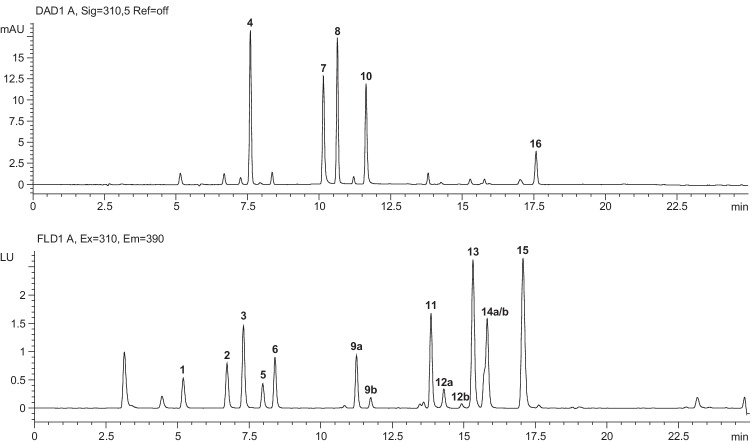
LC-DAD/FLD chromatograms (DAD: 310 nm, FLD: excitation 310 nm, emission 390 nm) showing baseline separation of all of the rodenticides (coumafuryl (**1**), warfarin (**2**), phenprocoumon (**3**), acenocoumarol (**4**), coumatetralyl (**5**), coumachlor (**6**), diphacinone (**7**), dicoumarol (**8**), tioclomarol (*cis*: **9a**, *trans*: **9b**), chlorophacinone (**10**), ferulenol (**11**), bromadiolone (*cis*: **12a**, *trans*: **12b**), difenacoum (**13**), flocoumafen (*cis*: **14a**, *trans*: **14b**), brodifacoum (**15**), difethialone (**16**); 0.01 µg/mL: **5**; 0.1 µg/mL: **1–3**, **6**, **9a/b**, **11–15**; 1 µg/mL: **4**, **7**, **8**, **10**, **16**) by ion-pair chromatography using a biphenyl column (150 mm × 4.6 mm, 2.6 µm) and gradient elution (solvent A: 10 mmol/L acetic acid/DIPEA in water, solvent B: 10 mmol/L acetic acid/DIPEA in methanol, flow rate: 0.7 mL/min, min/% B: 0/48, 10/78, 25/98, 30/48, 40 °C)

In a first step, we decided to replace commonly used tetrabutylammonium phosphate by volatile substitutes. This would maintain chromatographic conditions transferable to LC–MS/MS, e.g., for confirmatory analysis. Preliminary attempts using triethylammonium acetate (obtained in situ from equimolar amounts of triethylamine and acetic acid) already showed improved retention times and peak shapes. By switching to diisopropylethylamine (DIPEA) and adjustment to a 10 mmol/L amount-of-substance concentration, optimal conditions for baseline separation were finally achieved. Robust and sensitive detection of all rodenticides was subsequently ensured by careful selection of suitable absorption and/or emission wavelengths from UV/Vis (**4**, **7**, **8**, **10**, **16**) and fluorescence spectra (**1**–**3**, **5**, **6**, **9**, **11**–**15**) recorded using the LC-DAD/FLD system (for details and spectra, see Supplementary Information). An initial baseline drift observed when monitoring the DAD signal at 280 nm was successfully removed by a slight shift to 310 nm. Figure 2 shows a representative set of chromatograms with a minimum peak resolution *R*_S_ of 3.6 indicating full baseline separation of all sixteen rodenticides.

### In-house validation

**Fig. 3:**
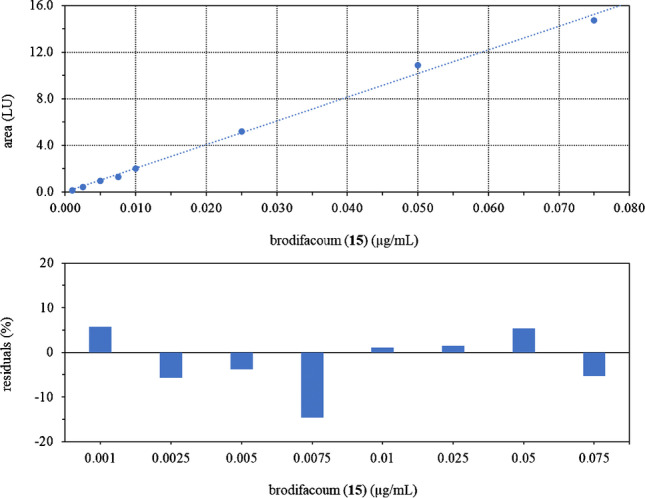
Calibration curve obtained by LC-FLD analysis of brodifacoum (15) following linear regression (top) and residual analysis (bottom) with a minimum requirement of *r*^2^ ≥ 0.99 and residuals within ± 20% of the nominal value without any observable trend

Suitability of the developed sample preparation and analysis procedure for routine application was confirmed by an in-house validation study. Based on an initial screening of detection sensitivity linearity was evaluated by linear regression and residual analysis in the range of 0.0001 to 100 µg/mL in eluent providing appropriate calibration curves, where *r*^2^ was always acceptable (≥ 0.99) and back-calculation of calibrators revealed residuals within ± 20% of the nominal value without any observable trend (Fig. 3, Table 2). Limits of detection (LOD) and quantification (LOQ) were subsequently derived following current EU and international guidance documents by tenfold analysis of blank wheat flour, which was selected as the validation matrix because a contamination of grain or cereals was considered a reasonable scenario [23]. Table 3 summarizes the validation data obtained with and without applying an optional, post-extraction solvent exchange, which may improve chromatographic resolution but in general is considered omissible as the LODs and LOQs obtained without this additional step are already adequate for screening purposes. Even the LOQs of **4**, **7**, **8**, **10** and **16** obtained by LC-DAD analysis in higher ppb range are still low enough to reveal a contamination only effective after repeated intake or as a result of a failed attempt.

**Table 2:** Linear working ranges observed in eluent as well as limits of detection (LOD) and quantification (LOQ) obtained from blank matrix analysis following the developed protocol (wheat flour, *n* = 10)

Rodenticide	Working range (µg/mL)	LOD (µg/kg)	LOQ (µg/kg)
Coumafuryl (**1**)	0.001–0.1	18.5	61.2
Warfarin (**2**)	0.001–0.1	5.9	19.5
Phenprocoumon (**3**)	0.0025–0.1	2.8	9.2
Acenocoumarol (**4**)	0.01–1.0	53.5	177
Coumatetralyl (**5**)	0.00025–0.01	1.5	5.0
Coumachlor (**6**)	0.001–0.1	2.6	8.7
Diphacinone (**7**)	0.01–1.0	48.3	159
Dicoumarol (**8**)	0.025–0.25	284	937
Tioclomarol (**9**)	0.001–0.1	5.8	19.2
Chlorophacinone (**10**)	0.025–1.0	163	538
Ferulenol (**11**)	0.0025–0.1	1.9	6.3
Bromadiolone (**12**)	0.0025–0.1	28.5	94.1
Difenacoum (**13**)	0.0025–0.1	6.0	19.7
Flocoumafen (**14**)	0.001–0.1	7.1	23.5
Brodifacoum (**15**)	0.001–0.1	3.1	10.2
Difethialone (**16**)	0.01–1.0	45.2	149

**Table 3:** Bias (mean recovery, %) and repeatability (relative standard deviation, %) rates obtained from blank matrix samples spiked to the determined LOQ of each rodenticide. Experiments were carried out with and without applying an additional solvent exchange to evaluate sufficient chromatographic stability when omitting this time-consuming, additional step (*n* = 8)

Rodenticide	Without solvent exchange		With solvent exchange	
	Bias (%)	Repeatability (%)	Bias (%)	Repeatability (%)
Coumafuryl (**1**)	98	4.1	85	9.6
Warfarin (**2**)	116	2.7	104	3.7
Phenprocoumon (**3**)	105	15.7	77	9.0
Acenocoumarol (**4**)	105	2.3	102	1.8
Coumatetralyl (**5**)	106	18.8	103	5.1
Coumachlor (**6**)	141	8.2	111	4.4
Diphacinone (**7**)	111	10.7	97	17.1
Dicoumarol (**8**)	92	7.4	87	7.3
Tioclomarol (**9**)	120	10.0	95	8.4
Chlorophacinone (**10**)	69	9.0	95	2.8
Ferulenol (**11**)	118	11.1	113	3.4
Bromadiolone (**12**)	110	18.1	92	11.3
Difenacoum (**13**)	97	2.6	97	5.2
Flocoumafen (**14**)	96	3.1	99	2.7
Brodifacoum (**15**)	77	12.4	117	8.9
Difethialone (**16**)	93	18.0	98	6.7

For example, the most potent rodenticide included in this study, brodifacoum, shows an oral LD_50_ in rats of 0.16 mg/kg body weight. Taking a safety factor of 100 for interspecies extrapolation and interindividual variability into account [35], a possibly still harmful dose of 1.6 µg/kg body weight can be derived for humans, which corresponds for an average person of 60 to 80 kg body weight to a total intake of 100 to 130 µg brodifacoum. Now, assuming a worst-case scenario, where all cereal-based products consumed by an average adult (EU: approximately 220 g/day [36]) are produced from the same poisoned raw material, the mass concentration of brodifacoum posing a serious threat would add up to 600 µg/kg. The determined LOQ of brodifacoum is 10 µg/kg and thus 60 times lower than this critical concentration and in good agreement with other reports (Table 1). As a second example, dicoumarol, e.g., the rodenticide showing the highest LOD of 284 µg/kg, may be considered, which has an oral LD_50_ in rats of 250 mg/kg. Considering all above-mentioned factors, the mass concentration of dicoumarol posing a potential threat to an average adult will be 680 mg/kg, which is more than 2000 times higher than the observed LOD. Sensitivity of the developed method was therefore judged satisfactory.

As pointed out above, we decided to skip a time-consuming, additional solvent exchange. Recovery as a measure of bias was therefore only evaluated applying each rodenticide’s LOQ obtained without this step, but with and without solvent exchange for comparison. Recovery rates were observed between 69 to 141% (without solvent exchange) and 77 to 117% (with solvent exchange) and are in good agreement with EU and international requirements for comparable concentration ranges, e.g., 60 to 130% for T-2 and HT-2 toxin (Commission Regulation (EC) No. 401/2006). Repeatability as determined by relative standard deviations from the eightfold recovery experiments with or without solvent exchange varied between 1.8 and 17.1% or 2.3 and 18.1%, respectively. Compared to the requirements for the detection of T-2 and HT-2 toxin, e.g., 30 or 40% above or below 250 ppb (Commission Regulation (EC) No. 401/2006), the overall repeatability observed below 20% corroborates the sufficient reliability of the developed screening method.

### Proof-of-concept scenario

To evaluate the applicability and robustness of the method in an improvised assault scenario, a rat poisoning bait of unknown composition was acquired from a local farmer. Solid rat poisoning baits are usually limited to a maximum content of 0.005% active ingredient, e.g., for brodifacoum set by Commission Implementing Regulation (EU) No. 2017/1381. It was ground to small pieces and mixed with wheat flour to obtain a sample mass concentration of approximately 5 to 10 mg/kg, which is expected toxic when used for example for preparation of pastries. Although the bait was dyed blue and the resulting mixture still had a blue shade, a sample of the contaminated flour was subjected to analysis to check if the dye or other components of the bait would interfere with analysis.

**Fig. 4:**
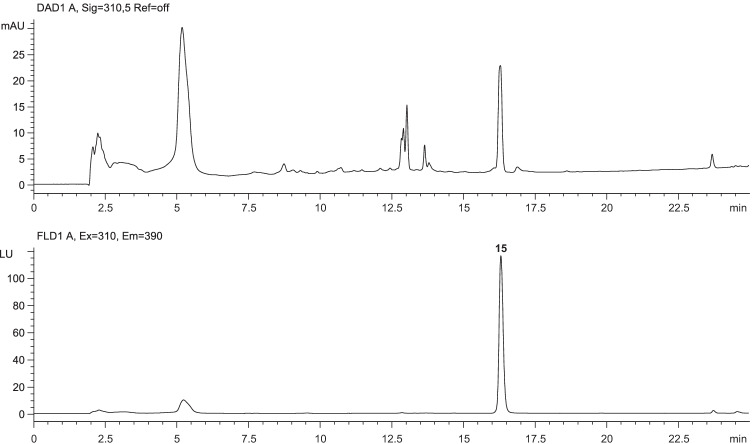
LC-DAD/FLD chromatograms obtained from a wheat flour sample (5 g) spiked with a commercial rat poisoning bait (1 g) to simulate intentional contamination. Brodifacoum (**15**) was unequivocally detected at a mass concentration of approximately 48 mg/kg with respect to the bait (for chromatographic details, see Fig. 2)

Fortunately, the highly water-soluble dye was visually removed already by extraction and no further interfering signals were observed during chromatography. The corresponding chromatograms (Fig. 4) showed a distinct signal for brodifacoum with a mean mass concentration in the rat poisoning bait of 48 mg/kg (*n* = 2). This finding is in good agreement with the general assumption that rat poisoning baits will be manufactured most effectively by using the maximum allowed legal limit of the corresponding rodenticide, i.e., 50 mg/kg. The findings of this assessment corroborate the very satisfying reliability of the developed analytical method for the screening of rodenticides in intentionally contaminated foodstuffs.

## Conclusion

Food defence measures have gained increased attention as a necessary supplement to established food safety provisions as the contamination of food or water supplies out of terroristic motivation appears nowadays more likely. This also holds true for military field operations, where troops often rely on local food or water supplies. The LC-DAD/FLD method reported herein offers a fast, simple, sensitive and robust procedure to deployed laboratories that allows for the screening of sixteen rodenticides. It may not only be applied for food defence but will also provide a valuable contribution to food safety in production areas exposed to rodenticides. The chromatographic conditions were carefully optimized taking compatibility with mass spectrometric detection into account, which might serve for confirmation of positive screening results.

## Supplementary Information

Below is the link to the electronic supplementary material.

Supplementary file1 (PDF 549 KB)
