# Supplementary material for: Methods in food defence: a simple and robust screening for 16 rodenticides by LC-DAD/FLD following QuEChERS–based extraction
Source: Anal Bioanal Chem. 2022 Jun 17;414(19):5829–36. doi: 10.1007/s00216-022-04145-w (PMC9293804; doi:10.1007/s00216-022-04145-w)

Supplementary Information

Methods in food defence: a simple and robust screening for 16 rodenticides by LC-DAD/FLD following QuEChERS-based extraction

Georg Menacher,[1] Benedikt Masberg,[1] Paul W. Elsinghorst[1,2,3]

[1] Central Institute of the Bundeswehr Medical Service Munich, 85748 Garching, Germany

[2] Pharmaceutical Institute, University of Bonn, 53121 Bonn, Germany

[3] Institute of Nutrition and Food Sciences, University of Bonn, 53115 Bonn, Germany

Spectral Measurements S2

UV/Vis and Fluorescence Spectra of **1-16** S3–S18

Spectral Measurements UV/Vis as well as fluorescence emission spectra were recorded using a standard restriction capillary (injection volume: 100 µL, flow rate: 0.3 mL/min) in combination with the diode array and fluorescence detectors of the liquid chromatography system (Agilent Infinity 1200, Agilent Technologies, Waldbronn, Germany). UV/Vis spectra in the range of 200 to 500 nm were obtained first to derive a common excitation wavelength of 310 nm. Corresponding fluorescence emission spectra in the range of 305 to 500 nm were subsequently recorded for optimized detection.

To avoid detector saturation acenocoumarol, chlorophacinone, dicoumarol, diphacinone, and difethialone were analysed at 10 µg/mL and all other rodenticides at 1 µg/mL mass concentration each prepared from 1000 µg/mL stock solutions. To consider any influence of pH as well as buffer composition, all solvent mixtures applied during chromatographic method development were investigated (Table S1). Mixing of each buffer (20%) with methanol (80%) was followed by pH measurements to determine the pH under the given chromatographic conditions.

Table S1 Buffer compositions used for chromatographic development and spectral measurements.

**buffer**	**composition**
1	3.85 g ammonium acetate, 2 mL acetic acid and 2 mL triethylamine dissolved in 1 L water
2	10 mmol/L aqueous ammonium acetate, pH 4.7 adjusted using 1 mol/L hydrochloric acid
3	10 mmol/L aqueous ammonium acetate
4	10 mmol/L aqueous ammonium carbonate, pH 9.1 adjusted using 25% aqueous ammonia

Figure S1 UV/Vis (top) and fluorescence-emission (bottom) spectra of coumafuryl (**1**) in different buffer/methanol (20/80, *v*/*v*) mixtures: (a) buffer 1, pH 5.80; (b) buffer 2, pH 6.38; (c) buffer 3, pH 7.23; (d) buffer 4, pH 8.83.

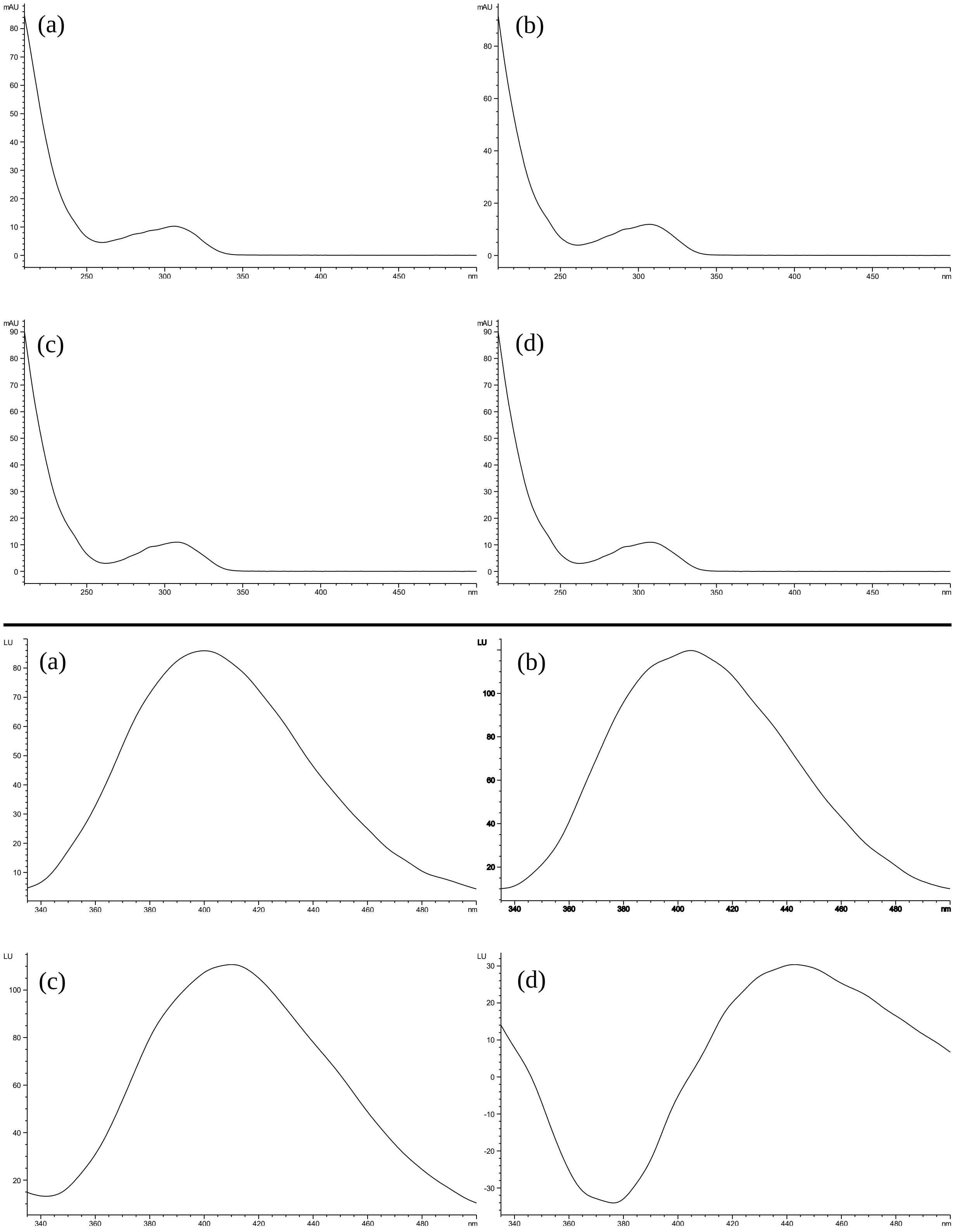

Figure S2 UV/Vis (top) and fluorescence-emission (bottom) spectra of warfarin (**2**) in different buffer/methanol (20/80, *v*/*v*) mixtures: (a) buffer 1, pH 5.80; (b) buffer 2, pH 6.38; (c) buffer 3, pH 7.23; (d) buffer 4, pH 8.83.

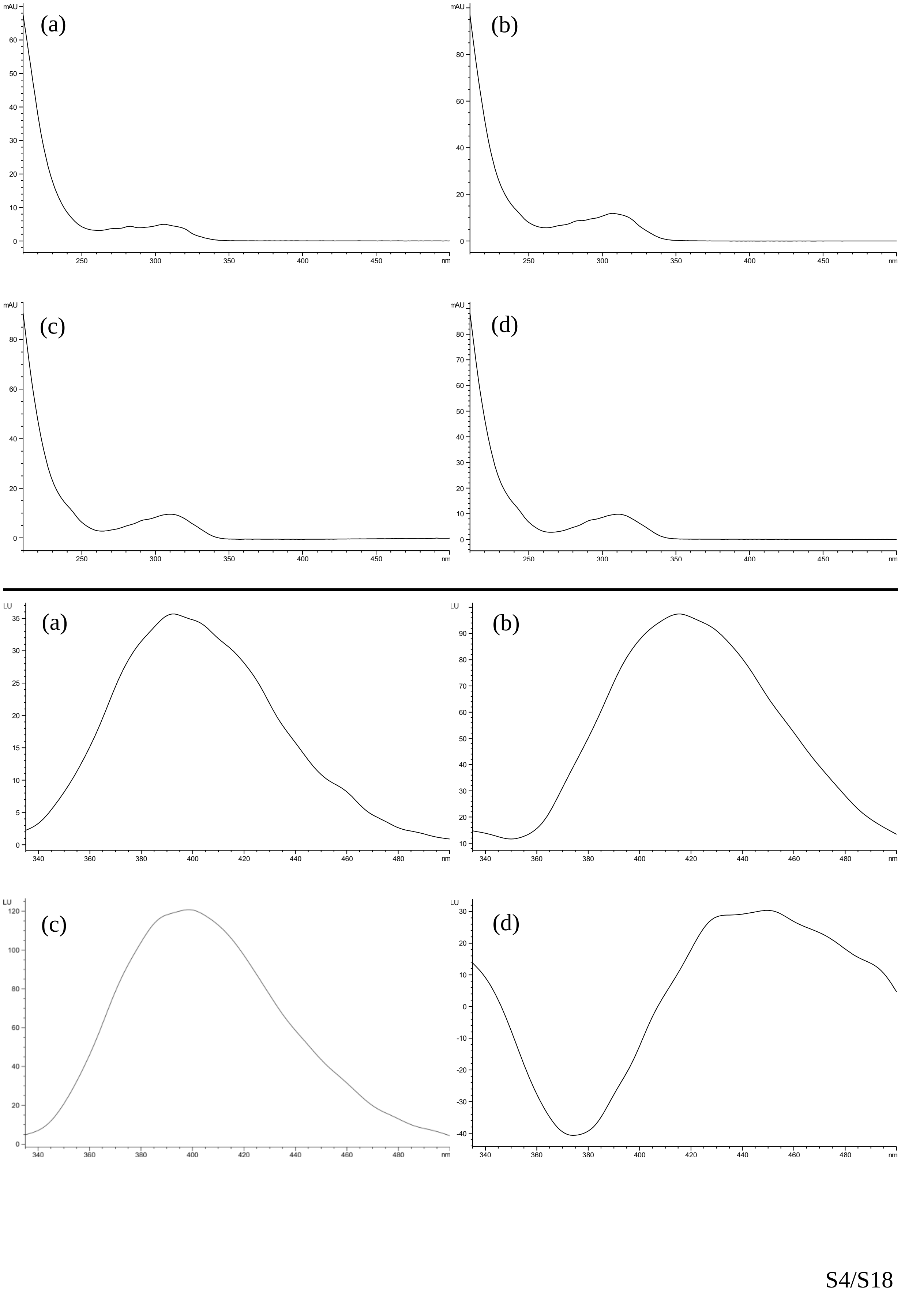

Figure S3 UV/Vis (top) and fluorescence-emission (bottom) spectra of phenprocoumon (**3**) in different buffer/methanol (20/80, *v*/*v*) mixtures: (a) buffer 1, pH 5.80; (b) buffer 2, pH 6.38; (c) buffer 3, pH 7.23; (d) buffer 4, pH 8.83.

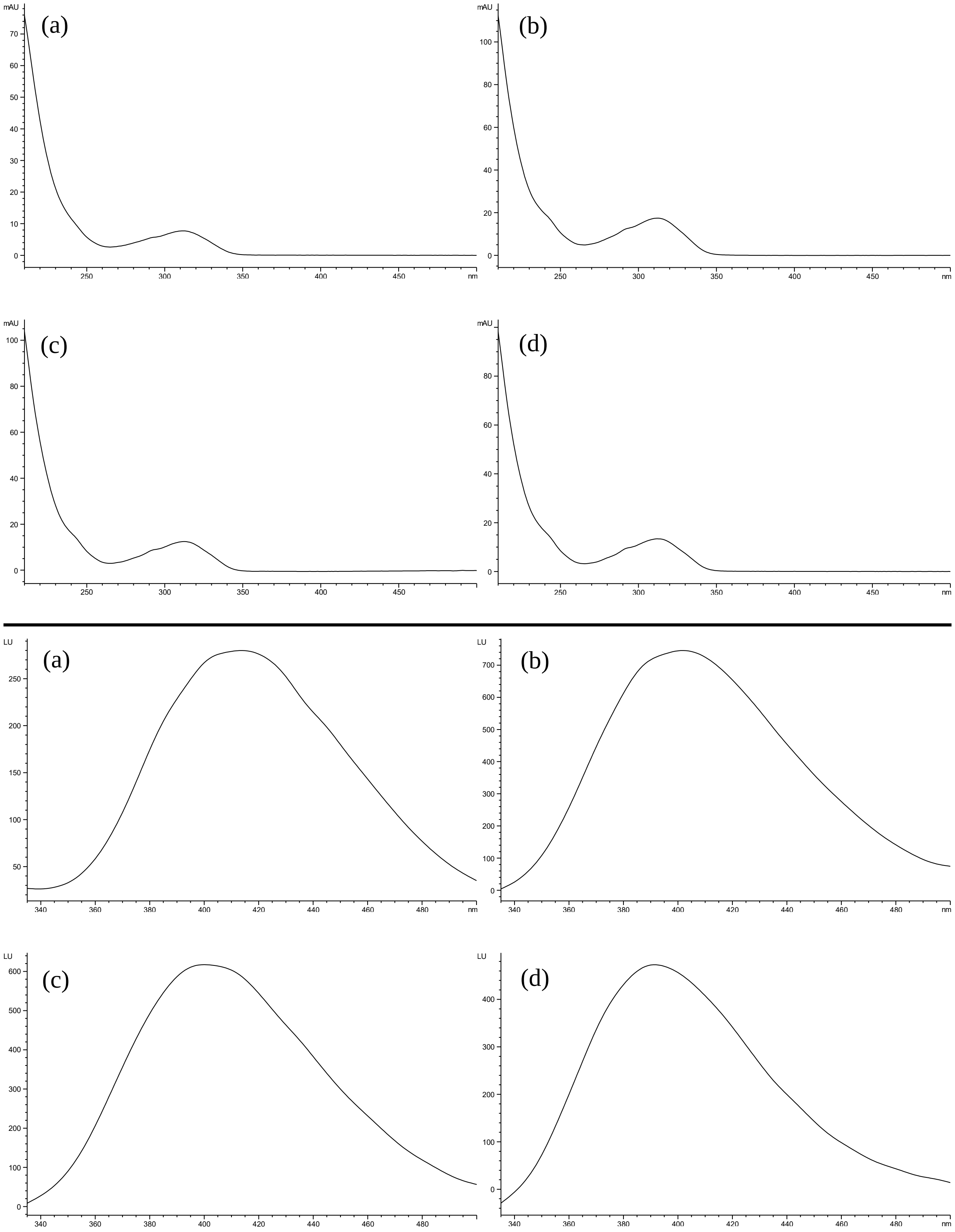

Figure S4 UV/Vis (top) and fluorescence-emission (bottom) spectra of acenocoumarol (**4**) in different buffer/methanol (20/80, *v*/*v*) mixtures: (a) buffer 1, pH 5.80; (b) buffer 2, pH 6.38; (c) buffer 3, pH 7.23; (d) buffer 4, pH 8.83.

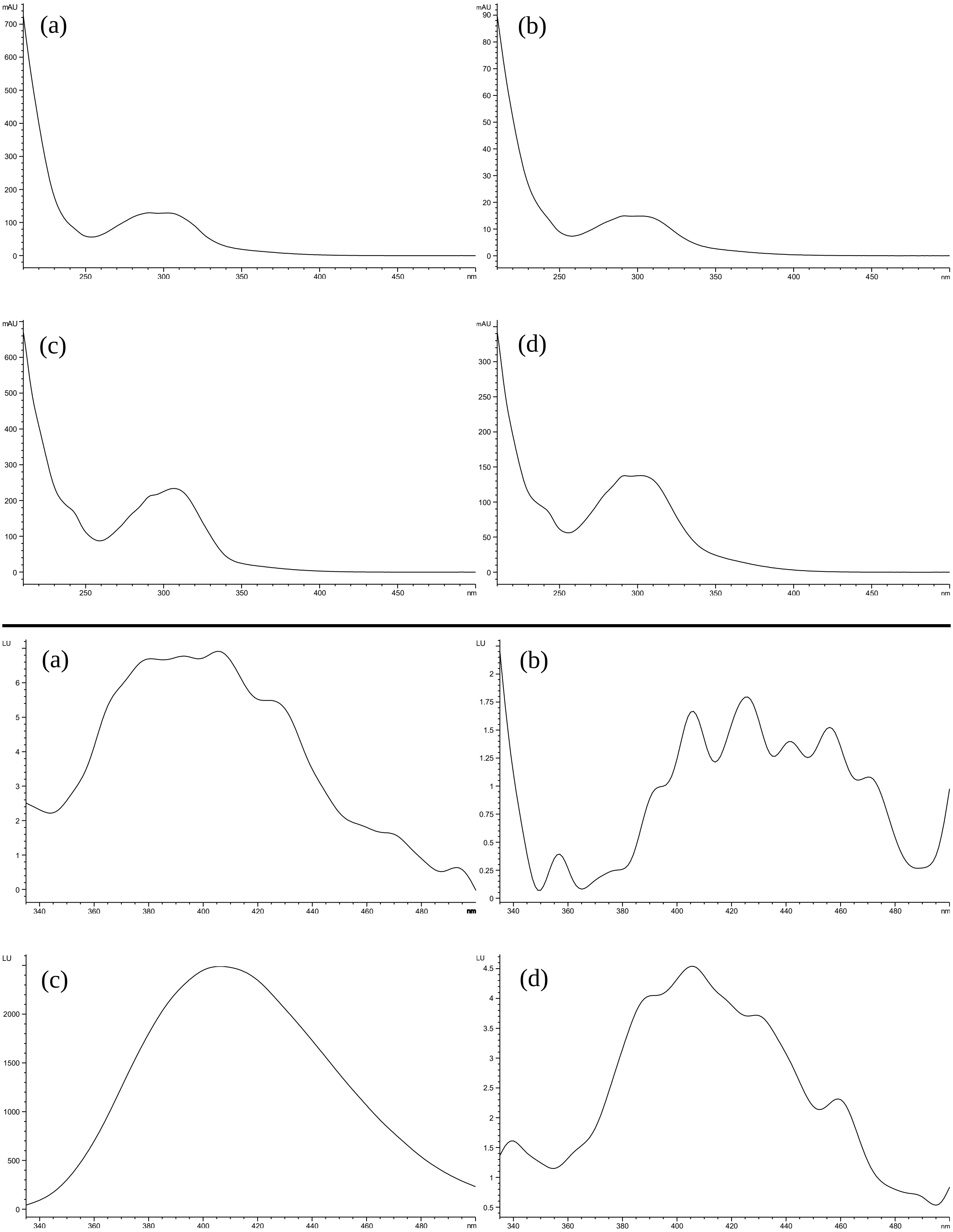

Figure S5 UV/Vis (top) and fluorescence-emission (bottom) spectra of coumatetralyl (**5**) in different buffer/methanol (20/80, *v*/*v*) mixtures: (a) buffer 1, pH 5.80; (b) buffer 2, pH 6.38; (c) buffer 3, pH 7.23; (d) buffer 4, pH 8.83.

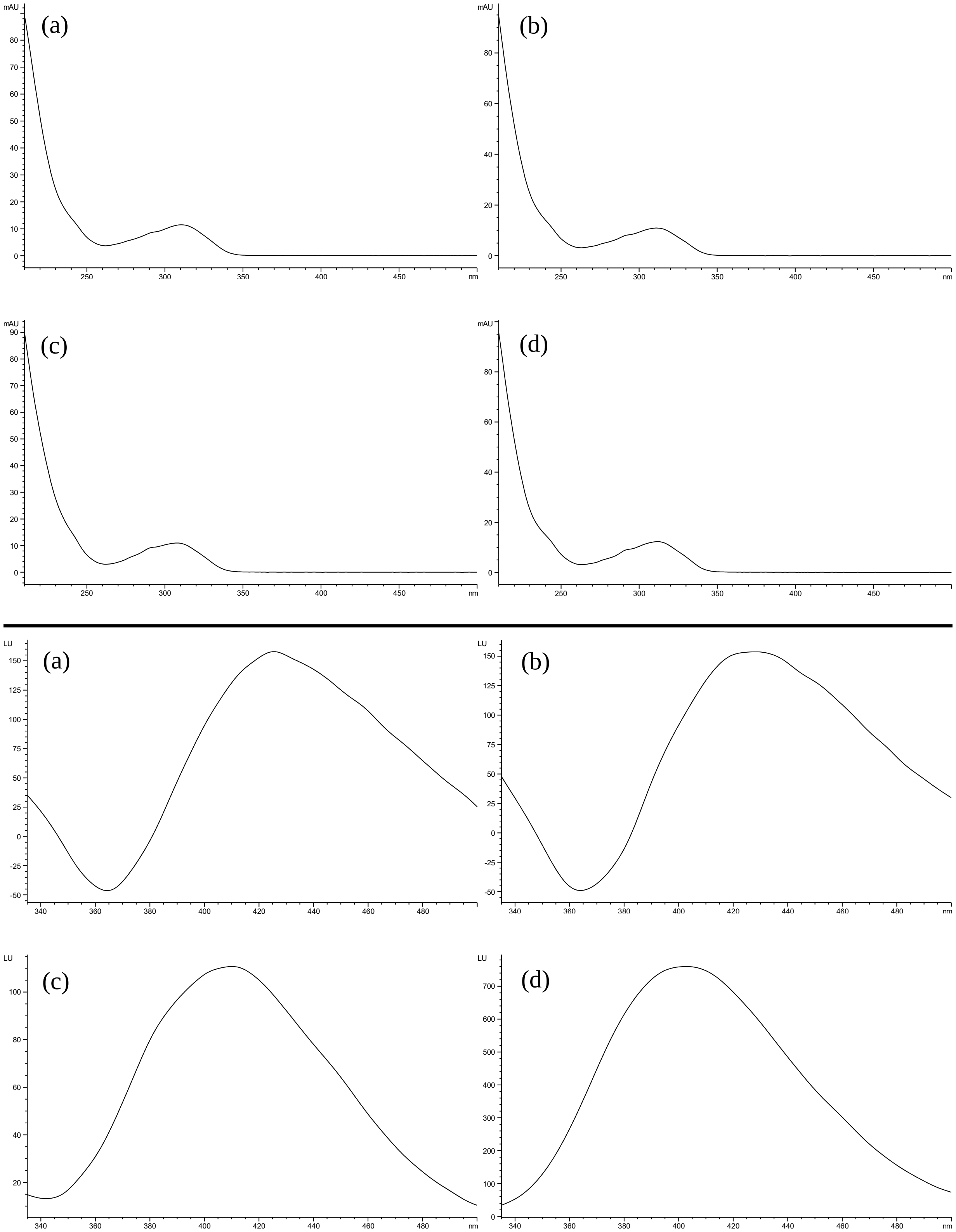

Figure S6 UV/Vis (top) and fluorescence-emission (bottom) spectra of coumachlor (**6**) in different buffer/methanol (20/80, *v*/*v*) mixtures: (a) buffer 1, pH 5.80; (b) buffer 2, pH 6.38; (c) buffer 3, pH 7.23; (d) buffer 4, pH 8.83.

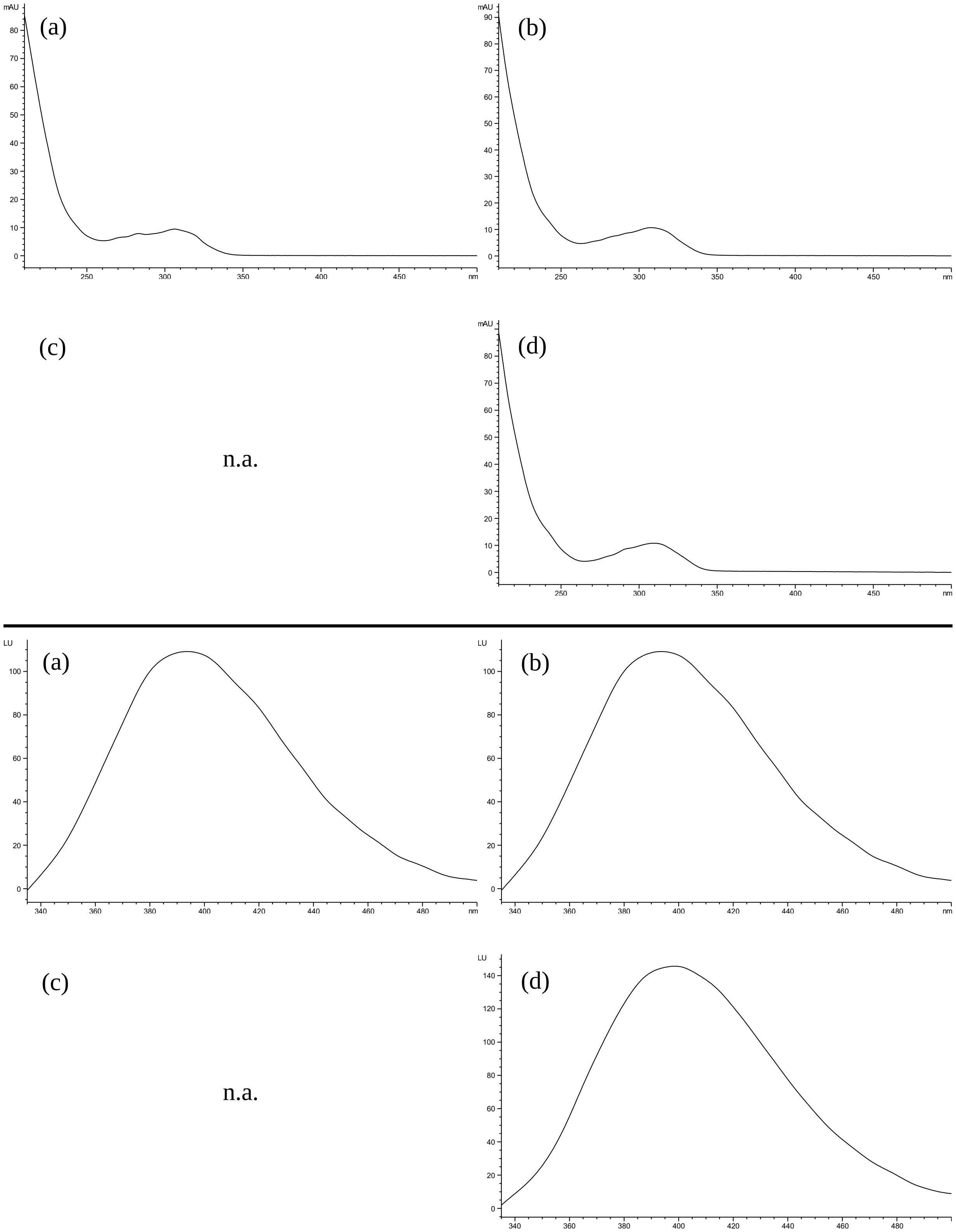

Figure S7 UV/Vis (top) and fluorescence-emission (bottom) spectra of diphacinone (**7**) in different buffer/methanol (20/80, *v*/*v*) mixtures: (a) buffer 1, pH 5.80; (b) buffer 2, pH 6.38; (c) buffer 3, pH 7.23; (d) buffer 4, pH 8.83.

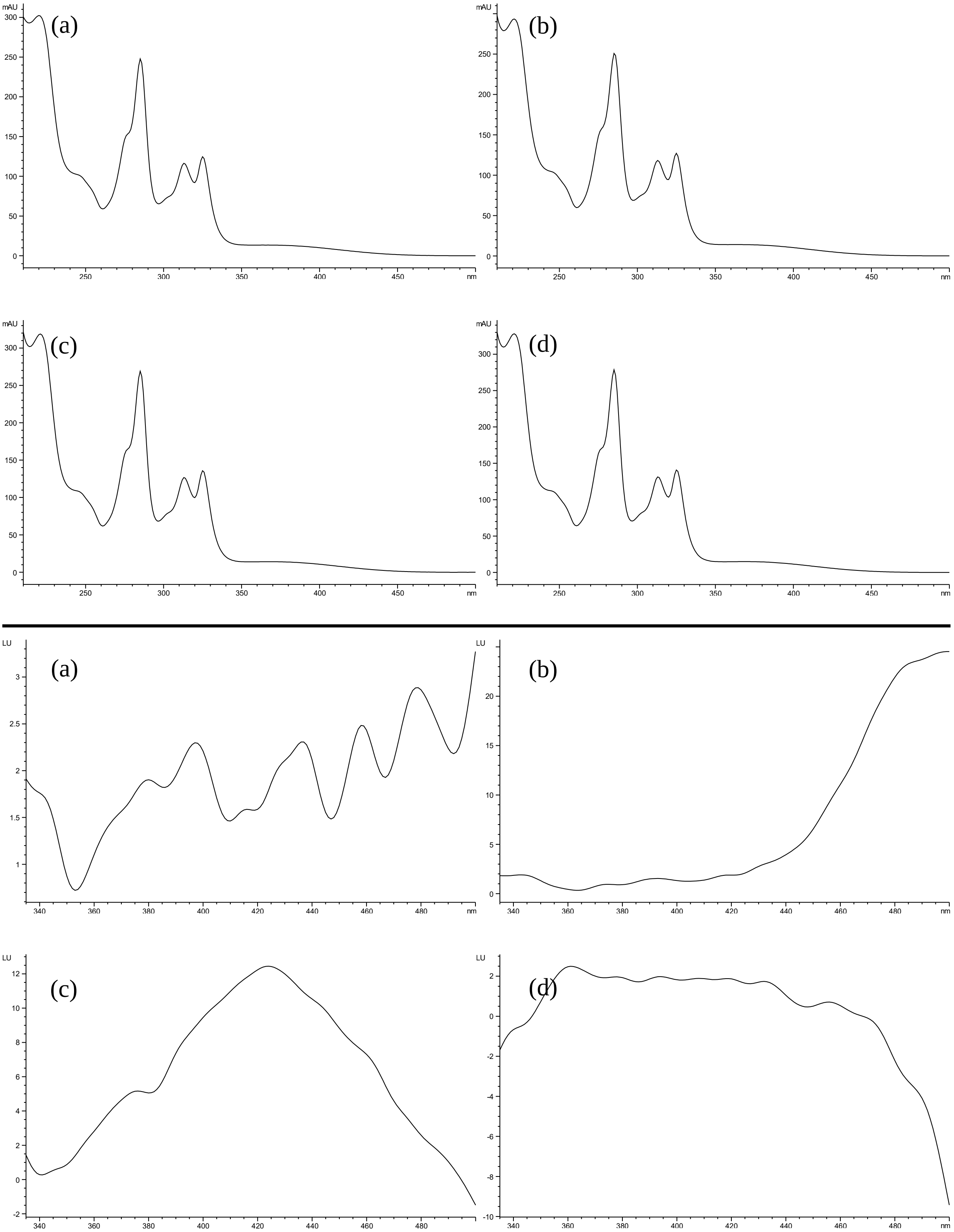

Figure S8 UV/Vis (top) and fluorescence-emission (bottom) spectra of dicoumarol (**8**) in different buffer/methanol (20/80, *v*/*v*) mixtures: (a) buffer 1, pH 5.80; (b) buffer 2, pH 6.38; (c) buffer 3, pH 7.23; (d) buffer 4, pH 8.83.

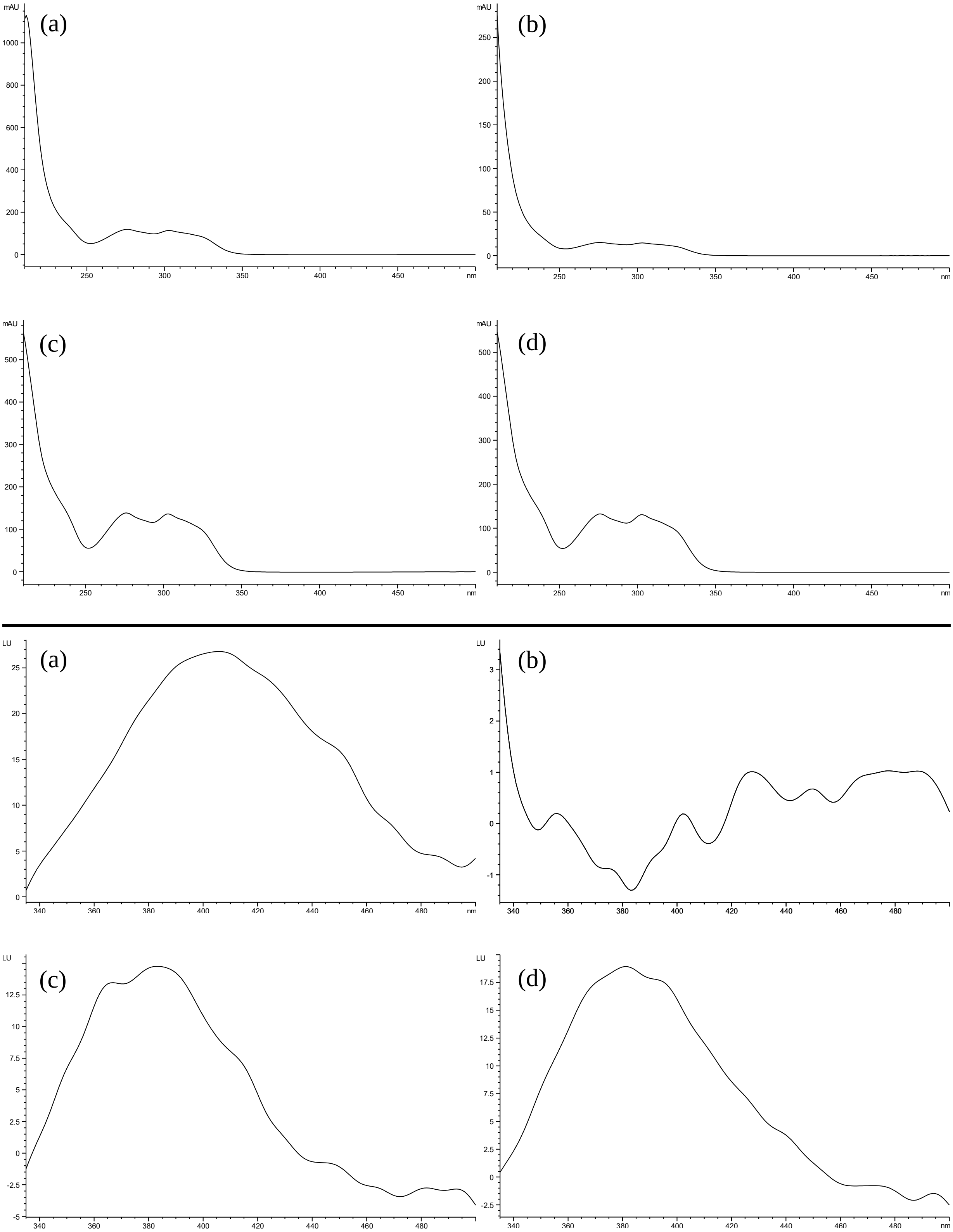

Figure S9 UV/Vis (top) and fluorescence-emission (bottom) spectra of tioclomarol (**9**) in different buffer/methanol (20/80, *v*/*v*) mixtures: (a) buffer 1, pH 5.80; (b) buffer 2, pH 6.38; (c) buffer 3, pH 7.23; (d) buffer 4, pH 8.83.

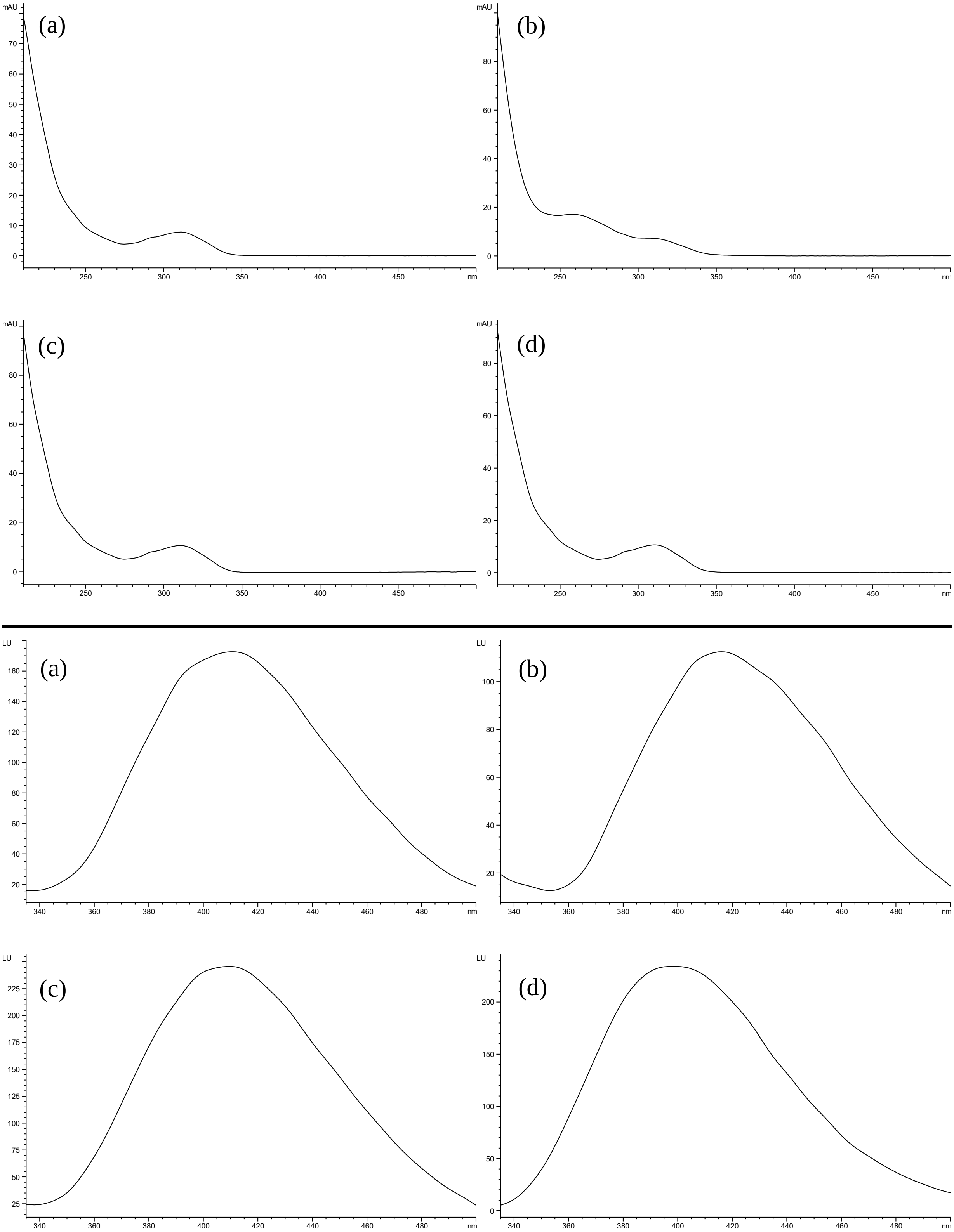

Figure S10 UV/Vis (top) and fluorescence-emission (bottom) spectra of chlorophacinone (**10**) in different buffer/methanol (20/80, *v*/*v*) mixtures: (a) buffer 1, pH 5.80; (b) buffer 2, pH 6.38; (c) buffer 3, pH 7.23; (d) buffer 4, pH 8.83.

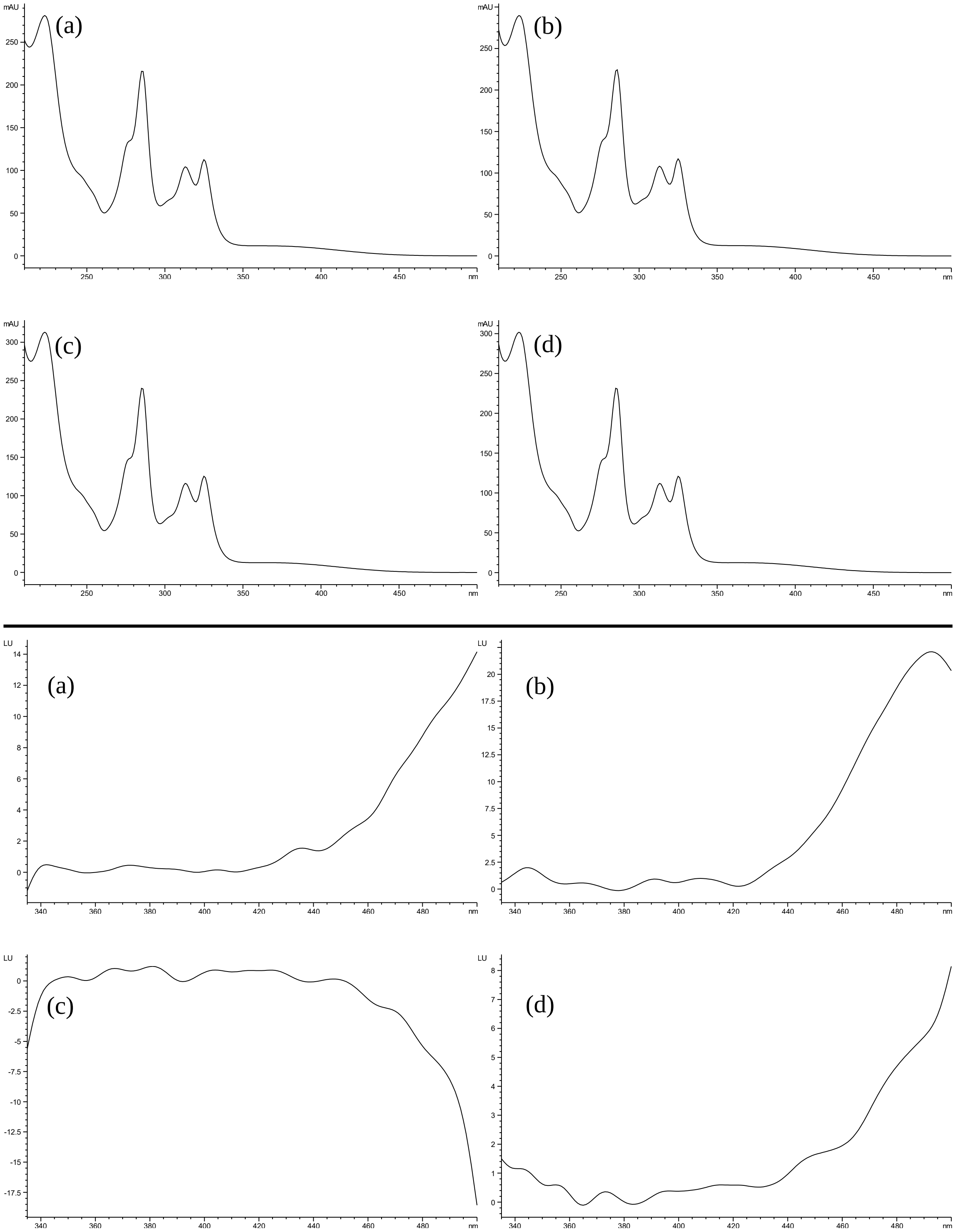

Figure S11 UV/Vis (top) and fluorescence-emission (bottom) spectra of ferulenol (**11**) in different buffer/methanol (20/80, *v*/*v*) mixtures: (a) buffer 1, pH 5.80; (b) buffer 2, pH 6.38; (c) buffer 3, pH 7.23; (d) buffer 4, pH 8.83.

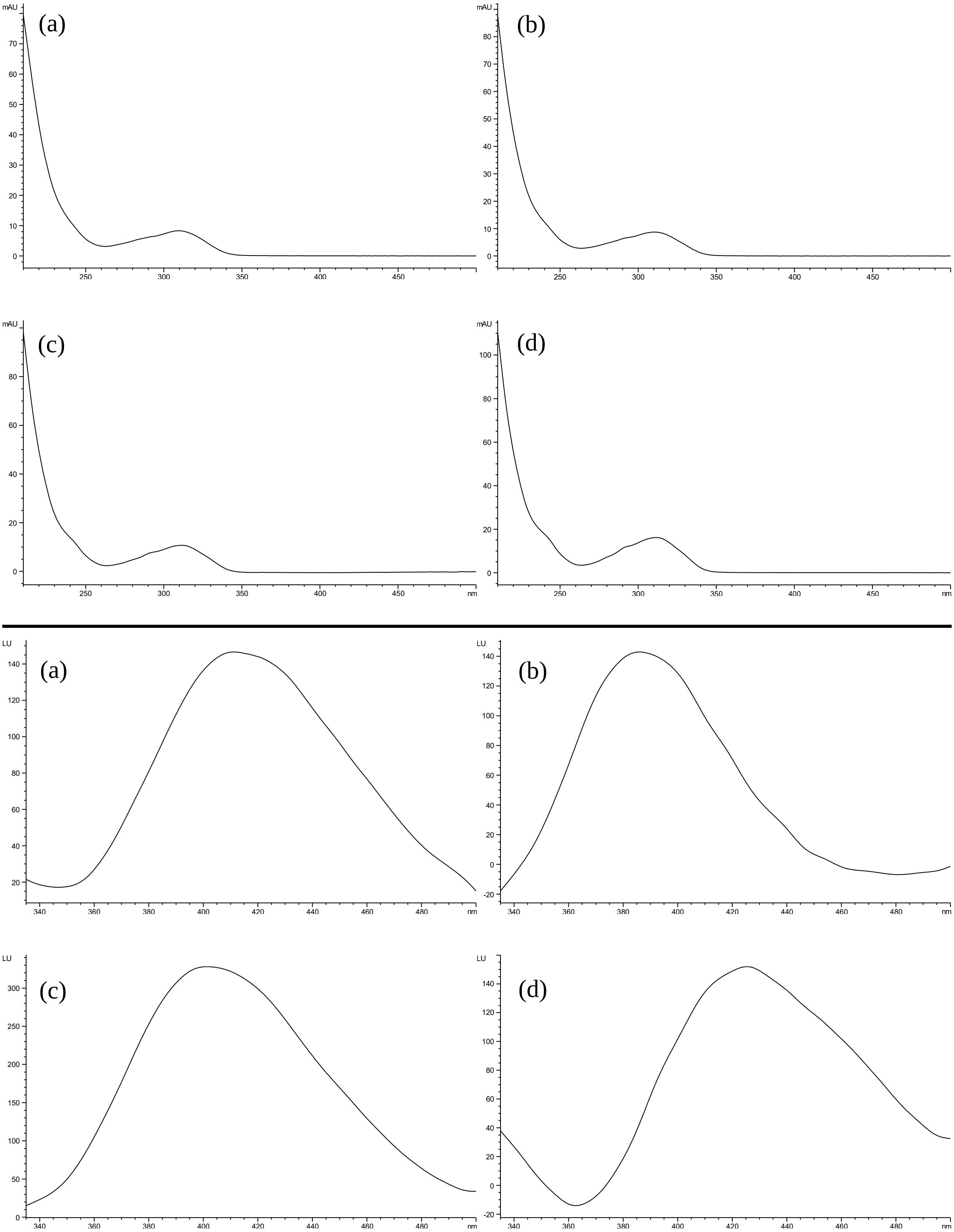

Figure S12 UV/Vis (top) and fluorescence-emission (bottom) spectra of bromadiolone (**12**) in different buffer/methanol (20/80, *v*/*v*) mixtures: (a) buffer 1, pH 5.80; (b) buffer 2, pH 6.38; (c) buffer 3, pH 7.23; (d) buffer 4, pH 8.83.

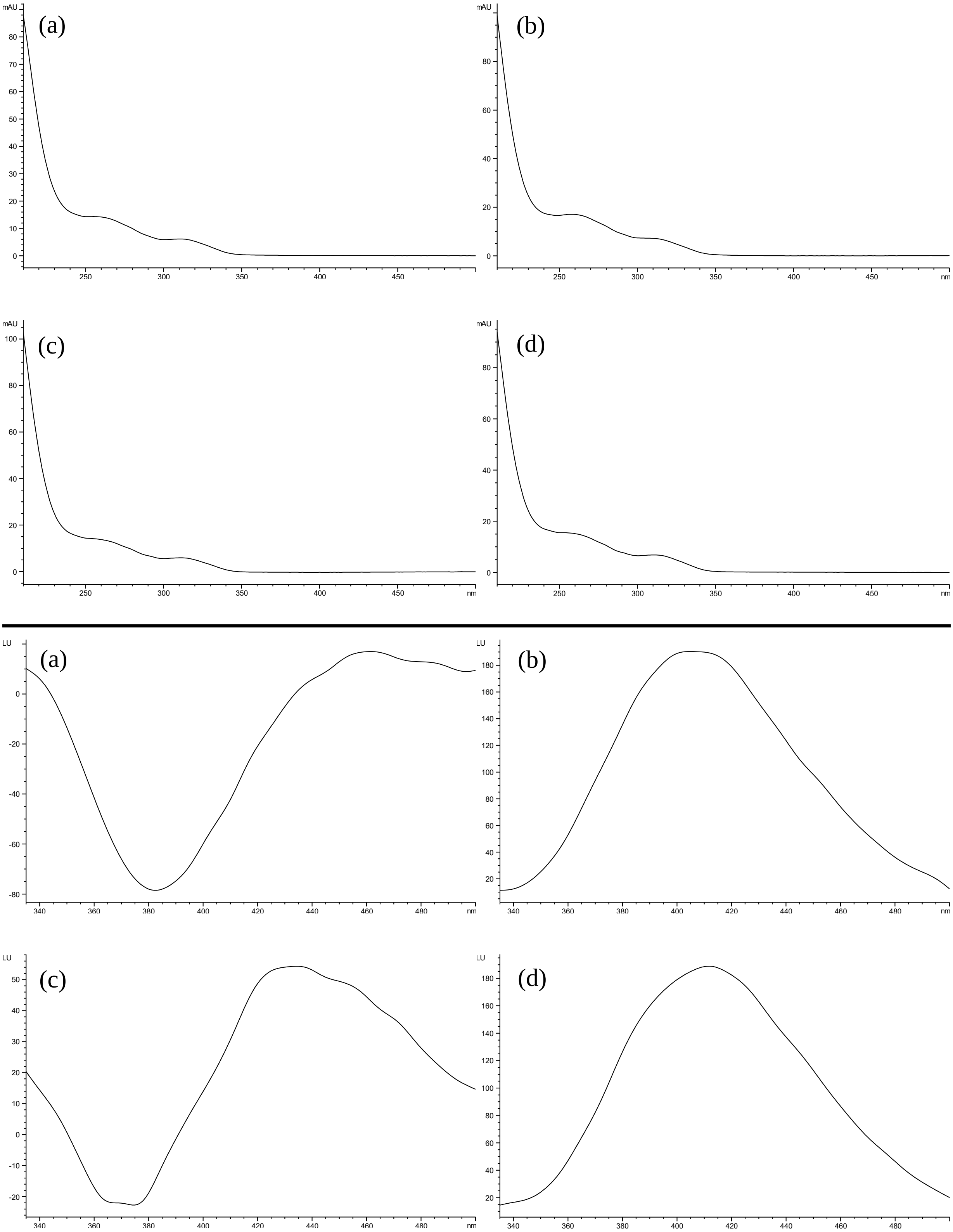

Figure S13 UV/Vis (top) and fluorescence-emission (bottom) spectra of difenacoum (**13**) in different buffer/methanol (20/80, *v*/*v*) mixtures: (a) buffer 1, pH 5.80; (b) buffer 2, pH 6.38; (c) buffer 3, pH 7.23; (d) buffer 4, pH 8.83.

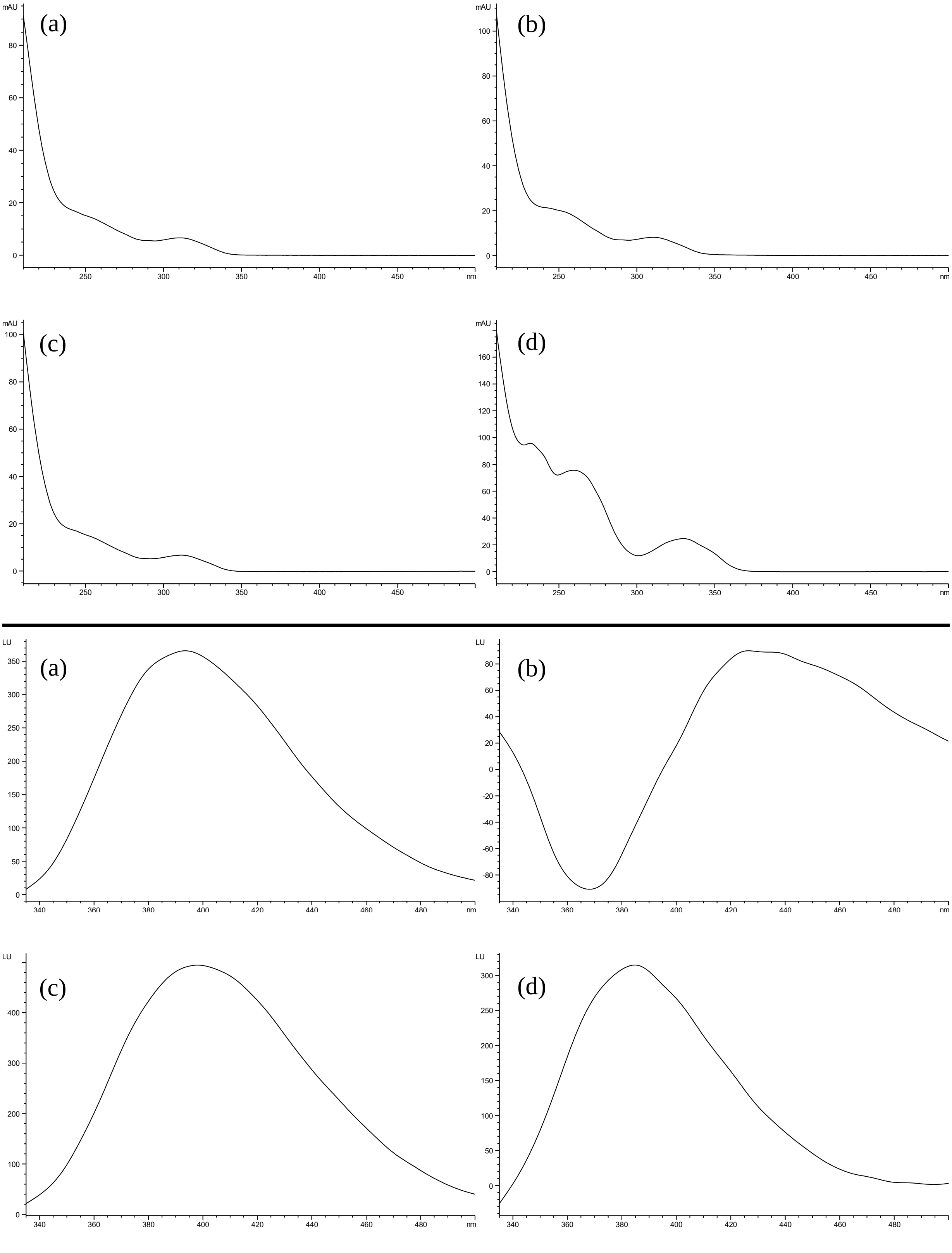

Figure S14 UV/Vis (top) and fluorescence-emission (bottom) spectra of flocoumafen (**14**) in different buffer/methanol (20/80, *v*/*v*) mixtures: (a) buffer 1, pH 5.80; (b) buffer 2, pH 6.38; (c) buffer 3, pH 7.23; (d) buffer 4, pH 8.83.

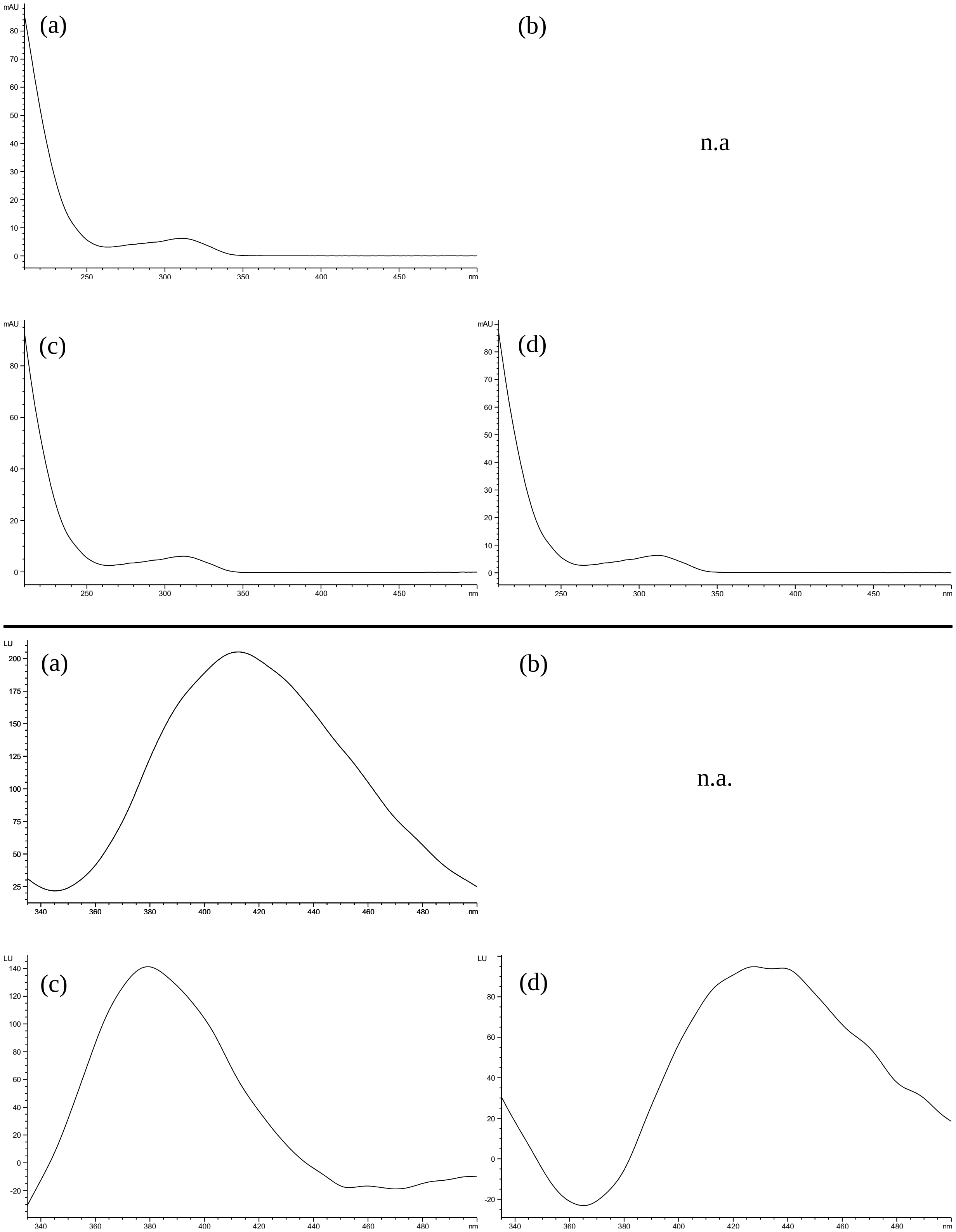

Figure S15 UV/Vis (top) and fluorescence-emission (bottom) spectra of brodifacoum (**15**) in different buffer/methanol (20/80, *v*/*v*) mixtures: (a) buffer 1, pH 5.80; (b) buffer 2, pH 6.38; (c) buffer 3, pH 7.23; (d) buffer 4, pH 8.83.

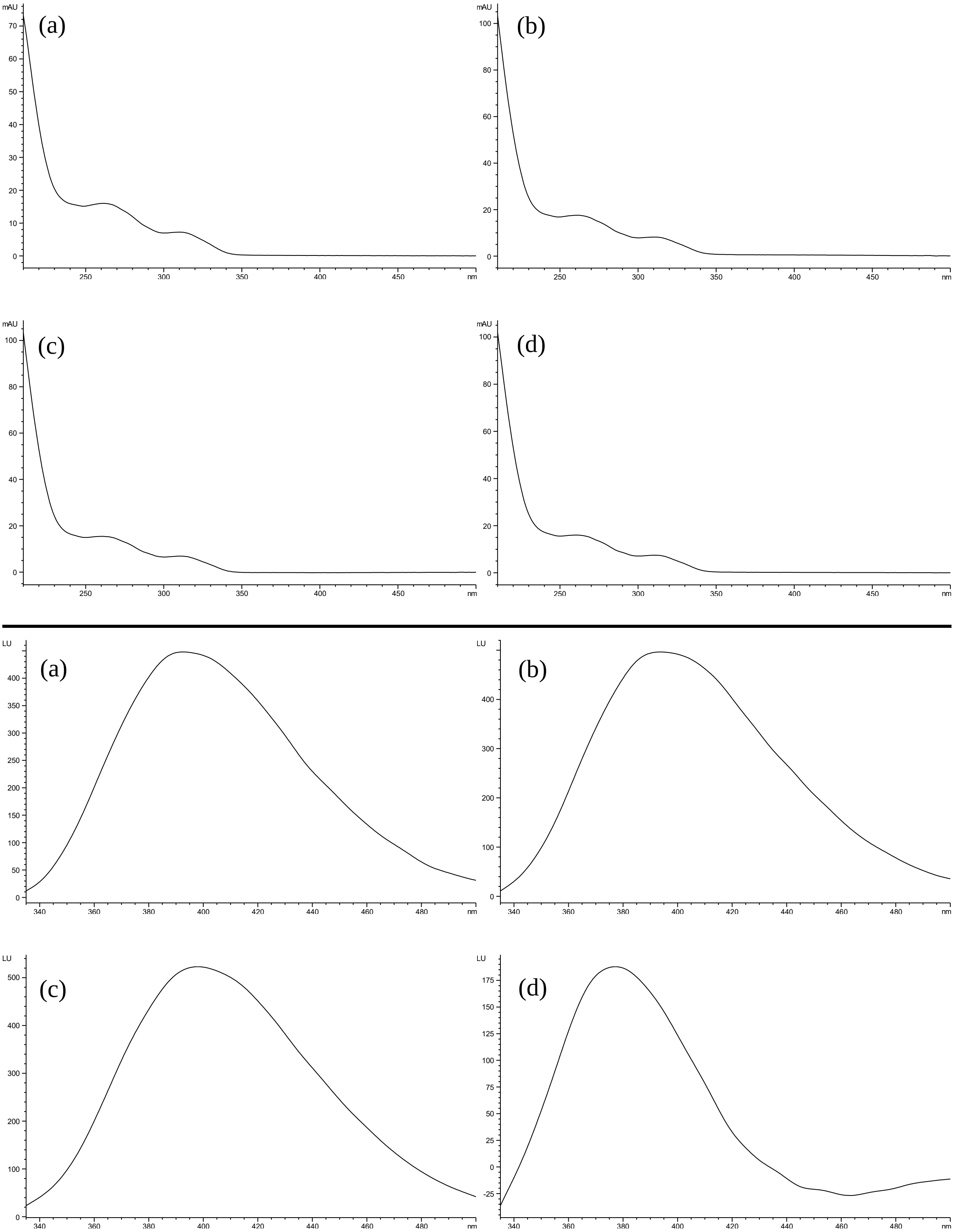

Figure S16 UV/Vis (top) and fluorescence-emission (bottom) spectra of difethialone (**16**) in different buffer/methanol (20/80, *v*/*v*) mixtures: (a) buffer 1, pH 5.80; (b) buffer 2, pH 6.38; (c) buffer 3, pH 7.23; (d) buffer 4, pH 8.83.

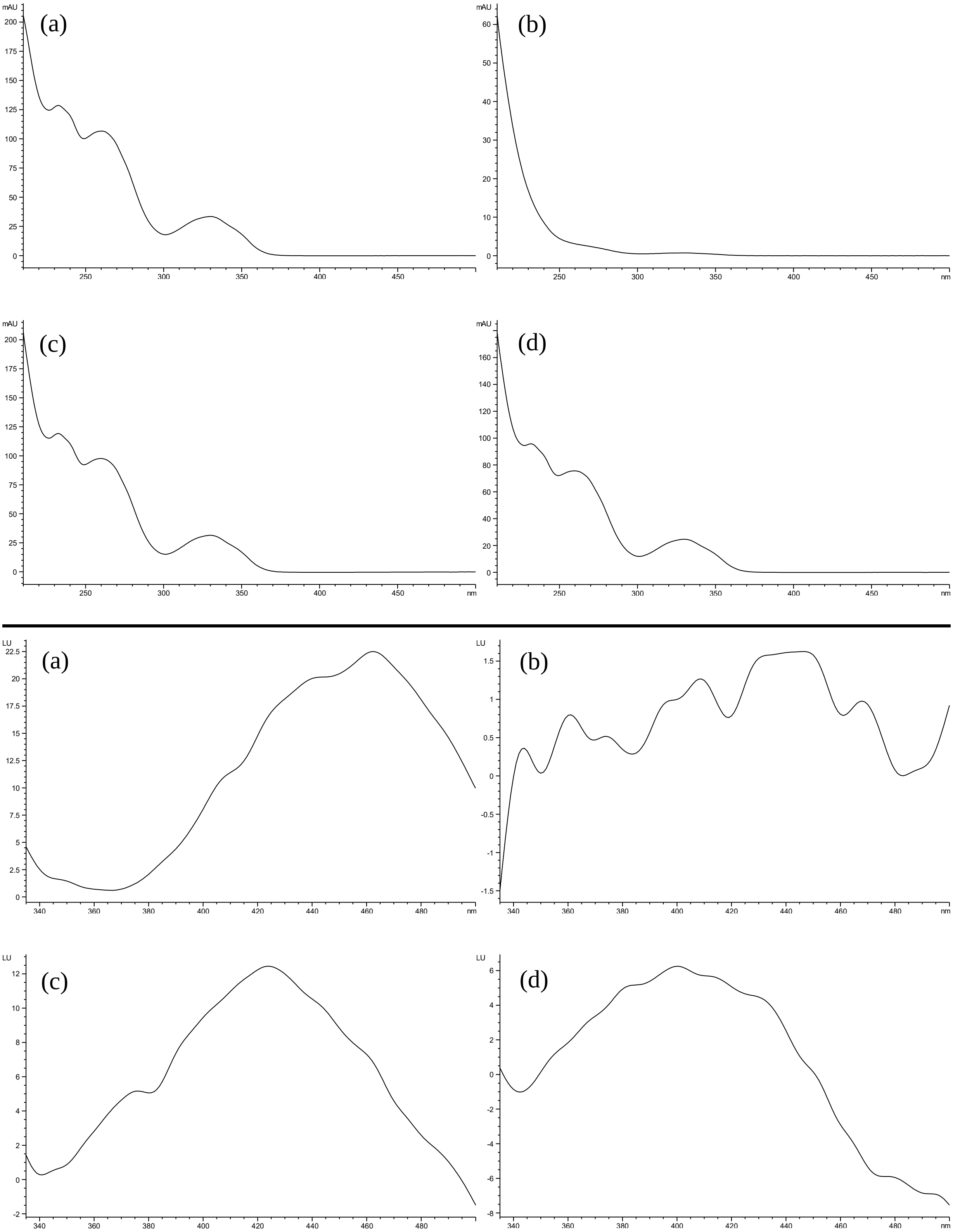

Supplement: Supplementary file 1 — Supplementary file1 (PDF 549 KB) [file 216_2022_4145_MOESM1_ESM.pdf]
